# Contrast Sensitivity Is Impaired in Suspected Primary Open-Angle Glaucoma Patients

**DOI:** 10.3390/brainsci14100993

**Published:** 2024-09-29

**Authors:** María Constanza Tripolone, Luis Alberto Issolio, Daniel Osvaldo Perez, Pablo Alejandro Barrionuevo

**Affiliations:** 1 Instituto de Investigación en Luz, Ambiente y Visión, Universidad Nacional de Tucumán—Consejo Nacional de Investigaciones Científicas y Técnicas, San Miguel de Tucumán T4000BLR, Argentina; mconstanzatripolone@gmail.com (M.C.T.);; 2 Departamento de Luminotecnia, Luz y Visión, Facultad de Ciencias Exactas y Tecnología, Universidad Nacional de Tucumán, San Miguel de Tucumán T4000BLR, Argentina; 3 Cátedra de Oftalmología, Facultad de Medicina, Universidad Nacional de Tucumán, San Miguel de Tucumán T4000BLR, Argentina; 4 Allgemeine Psychologie, Justus-Liebig-Universität Gießen, 35394 Gießen, Germany

**Keywords:** glaucoma, functional assessment, early detection, age, contrast sensitivity

## Abstract

Purpose: To assess spatial contrast sensitivity (CS) in suspected primary open-angle glaucoma (POAG) patients. Methods: CS was measured using sinusoidal gratings of 4 cycles/degree. First, foveal and peripheral CS were assessed in 34 suspected POAG patients and compared with 71 and 28 age-matched healthy individuals for foveal and peripheral conditions, respectively. Second, foveal CS was assessed in 34 early POAG patients age-matched with suspected POAG patients. Analyses were performed considering two age ranges: Under and Over 50 y.o. Correlations were evaluated between CS and clinical parameters. Diagnostic accuracy was also analyzed. Results: Peripheral CS was lower in older suspected POAG patients (23.4 ± 16.1) than the control group (39.1 ± 28.2) (*p* = 0.040). Foveal CS was reduced in suspected POAG participants (Under 50: 146.8 ± 63.3; *p* = 0.004. Over 50: 110.5 ± 65.0; *p* = 0.044) and in early POAG patients (Under 50: 141.2 ± 72.6; *p* = 0.002. Over 50: 80.2 ± 54.5 *p* < 0.001), both compared to the control group (Under 50: 213.5 ± 66.2. Over 50: 138.6 ± 71.7). CS was lower in early POAG than in POAG suspected in older patients (*p* = 0.042). Foveal CS was correlated with age (Early: *p* = 0.001. Suspect: *p* = 0.002) and with the cup–disc ratio only in early POAG patients (*p* < 0.001). Foveal CS had fair (AUC = 0.74) diagnostic accuracy for early POAG patients. Conclusions: CS in suspected POAG patients is lower than in healthy individuals. Our findings evidence the spatial vision loss before the onset of POAG.

## 1. Introduction

Primary open-angle glaucoma (POAG) is an optic neuropathy characterized by progressive damage of the retinal ganglion cells and the optic nerve, leading to irreversible visual deterioration, and in some cases, blindness [1]. Early diagnosis and appropriate treatment can prevent the progression of the disease, and consequently, improve the patient’s quality of life. Because POAG is a leading cause of blindness worldwide [2], it is important to detect and evaluate the changes produced at early stages, before an irreparable loss of vision occurs.

There is no precise standard method for detecting POAG at its earlier stages since patients generally do not present symptoms other than a gradual visual field loss [3,4,5]. Elevated intraocular pressure (IOP), older age, and a family history of glaucoma are some of the risk factors that indicate an increased likelihood of developing POAG [6,7]. Diagnostic testing for POAG includes structural and functional examination. Evaluation of the optic disc and retinal nerve fiber layer (RNFL) has improved with the development of imaging techniques, such as Optical Coherence Tomography, allowing for an objective structural assessment [8,9]. However, variability in healthy individuals makes identifying early damage caused by POAG difficult [5]. Standard Automated Perimetry has been a gold standard for evaluating functional deficits [10]. Indeed, visual field scores have been used to categorize the stage of glaucoma [11,12]. However, 30% to 50% of ganglion cells are damaged until a significant visual field loss is detected [13,14,15,16]. Thus, visual field tests are useful in more advanced stages of POAG, but not to detect early functional damage.

Contrast sensitivity (CS) measures the spatial response of the human visual system. It is calculated as the inverse of the minimum contrast (threshold) that can be detected by a person, stimulated with a grating pattern, and characterized by its spatial frequency expressed in cycles per degree [17,18]. CS has been measured to evaluate functional spatial vision loss in glaucoma patients [19]. Previous studies have found a reduced CS in POAG patients measured at the fovea [20,21,22,23,24,25,26,27]. Furthermore, a significant correlation was reported between CS and cup–disc ratio (CDR) [26], retinal nerve fiber layer thickness [20], Ganglion Cell/Inner Plexiform Layer Sector Thickness [28], and mean deviation (MD) visual field score [22,26,28]. Since visual field loss often starts in the periphery, CS was also measured outside the fovea. Peripheral CS sensitivity was reduced in POAG patients with early visual field loss [21,27,29]. Moreover, in ocular hypertension (OHT) patients, peripheral CS was reduced whereas foveal CS remained at normal values [25]. Although there is broad evidence that CS is reduced in moderate and advanced POAG, there is not enough information on the first stages, which are critical for early diagnosis.

On the other hand, there is further evidence that CS is associated with normal aging [29,30,31,32], caused by age changes in optical and neural factors. A significant CS difference was reported in healthy individuals between younger and older adults, indicating a cut-off value at the age of 50 years old [30,32]. For clinical purposes, the age of participants is crucial when comparing patients with healthy individuals. In this way, CS differences could be attributed to the disease and not to normal aging changes.

Knowing whether functional changes may indicate early impairment caused by POAG is particularly important. This study aims to evaluate CS changes in patients with suspected POAG. Two experiments were performed. In the first one, CS was assessed at different retinal positions and light levels in suspected POAG patients and healthy participants. In the second experiment, we investigated the informative value of CS in our best discrimination condition (obtained in Experiment 1) concerning the results of patients with early POAG. Foveal CS under photopic light conditions was measured in early POAG patients and compared with the results of suspected POAG patients. Analyses of CS were assessed in two age ranges, Under and Over 50 years old. Correlations between CS and clinical parameters in early and suspected POAG patients were evaluated. Finally, to assess the ability of CS to differentiate patients from healthy individuals, diagnostic accuracy analyses were performed

## 2. Materials and Methods

### 2.1. General Methods

#### 2.1.1. Participants

Patients with early POAG and suspected POAG were recruited from the Ophthalmology Service of the National University of Tucumán, in the San Miguel de Tucumán city (Argentina). All participants underwent a complete eye examination including visual acuity (VA) (logMAR visual acuity chart “R” at 8 feet; Vector Vision, Greenville, OH, USA), IOP (Perkins tonometer; Haag-Streit, Köniz, Switzerland), slit-lamp biomicroscopy (Topcon SL-3C; Topcon Corporation, Tokyo, Japan), ophthalmoscopy (Topcon TRC 50EX Retinal Camera; Topcon Corporation, Tokyo, Japan), and visual field test (Program G1/TOP, Octopus 311; Haag-Streit, Köniz, Switzerland).

The diagnosis of POAG was established on the presence of glaucomatous optic disc (diffuse or focal narrowing, or notching, of the optic disc rim; disc hemorrhage; enlarged CDR; inter-eye asymmetry of CDR > 0.2) and correlated, confirmed, and reliable visual field defects [6], with a confirmed anterior chamber open-angle. POAG was classified based on the Octopus visual field staging system, reported by Mills and colleagues [12]. The early stage of POAG was defined as an MD score up to 4.4 and a value from 41 to 51 in the Bebie Curve where the lower confidence limit line intersects with the patient line. The diagnosis of suspected POAG was defined based on the presence of clinical findings and risk factors that indicate and increase the likelihood of developing POAG (optic disc rim changes, as described above, suspicious for glaucomatous damage; elevated IOP associated with normal appearance of the optic disc; and confirmed family history of POAG), without visual field defects (value above 51 in the Bebie Curve where the lower confidence limit line intersects with the patient line) [7,33,34]. Participants with other ocular pathology unrelated to POAG were excluded, including retinopathy or optic neuropathy.

Healthy control participants were recruited from a university cohort. Volunteers were selected to age-match with early and suspected POAG patients. Healthy participants had no ocular or systemic pathology, including any kind of retinal pathology or optic neuropathy, confirmed with a complete eye examination, as detailed above. Healthy individuals with a family history of POAG were excluded.

#### 2.1.2. Apparatus

A computer-based CS measurement system was used, previously described by Colombo and colleagues [35]. This system generates visual stimuli that were presented on a widescreen CRT monitor of 17″, modified with an attenuator to obtain a resolution of 13 bits, with about 3500 grey levels after gamma correction [36]. The stimuli consisted of achromatic sinusoidal gratings with a Michelson contrast between 0.002 and 1. The system allowed the selection of the stimulus spatial frequency within a wide range (1, 2, 4, 8, 12, and 24 cycles/degree). Each stimulus was presented at its nominal contrast during 500 ms and was temporally modulated with ascending and descending ramps of 250 ms, making the stimulus appear gradually. Also, it was spatially modulated by a Gaussian function, generating a Gabor patch.

The observer’s eye was placed 1.5 m from the monitor screen, resulting in a visual stimulus size of 4°. The patches were tilted 7 degrees from the horizontal and each of them was presented randomly in either a clockwise or counterclockwise direction. The system employs a discrimination task with a forced choice selection of two alternatives. Using a joystick with two buttons (one for each inclination), the observer must indicate the stimulus orientation, being forced to pick one of them. A modified adaptive psychophysical method based on the QUEST algorithm was used to determine the contrast threshold [37]. This algorithm makes a Bayesian inference of each response to establish the contrast of the following stimulus to be presented. Between 25 and 42 trials were required to determine the CS at a specific spatial frequency (the number of trials depends on the observer’s responses).

#### 2.1.3. Measurement Conditions

Foveal and peripheral CS were measured at a spatial frequency of 4 cycles/degree (c/d). This spatial frequency was selected based on a previous work [38], in which, at the photopic level, foveal CS was determined for spatial frequencies of 4 c/d and 8 c/d while, at the mesopic level, foveal and peripheral CS was determined for 4 c/d and 2 c/d, respectively. From this previous study, 4 c/d was found to be more adequate for evaluating photopic and mesopic CS than 8 c/d and 2 c/d, respectively. Foveal CS was measured at the photopic light level, with a stimulus mean luminance of 70 candela/metre^2^ (cd/m^2^), favoring the detection of this retinal region. Peripheral CS was measured with stimuli located on the inferonasal retina quadrant, at 9 degrees of eccentricity. This stimulus condition was achieved using a gaze fixation point (red LED) located 0.24 m from the stimulus center, on the 45° downwards diagonal at the opposite side of the eye tested. This quadrant was chosen considering that ganglion cell density is higher in the nasal retina than in other regions [39,40], and the inferior retina presents more vulnerability for glaucomatous damage [9,41]. Because rod density is higher in this retina area than in the fovea [42], peripheral CS was determined at the mesopic light level. Using a neutral filter placed in front of the screen monitor, the stimulus mean luminance was 0.5 cd/m^2^.

The test was conducted monocularly, covering the fellow eye with an eye patch. Both eyes were tested in all participants, in a single session. The participants were dark-adapted for 5 min before the session began. For the mesopic range, the participants were also 3 min adapted to the mesopic light level. Each measurement condition (foveal and peripheral) lasted a mean duration of 15 min. Data from eyes with a VA worse than 0.3 logMAR were excluded from the analysis, as well as data from eyes that could not detect the first 10 Gabor patches presented on the monitor.

#### 2.1.4. Data Analysis

The participants were grouped by age range: Under 50 Years Old (<50 y.o.) and Over 50 Years Old (≥50 y.o.). Statistical analysis was performed with Matlab software (MathWorks, Natick, MA, USA, https://www.mathworks.com/products/matlab.html), adopting a significance level of *p* ≤ 0.05. Since a two-eye experimental design was adopted [43], differences between groups were determined by nested mixed effects analysis of variance (ANOVA) [44]. Pearson correlations between CS and clinical parameters were calculated with Minitab software (Minitab Inc., State College, PA, USA, https://www.minitab.com/en-us/products/minitab/). Receiver operating characteristic (ROC) analyses were performed with MedCalc software (MedCalc Software Ltd., Ostend, Belgium, https://www.medcalc.org/) to determine the diagnostic accuracy of CS.

### 2.2. Specific Methods

#### 2.2.1. Experiment 1: Contrast Sensitivity in Suspected POAG Patients

The purpose of experiment 1 was to assess foveal and peripheral CS in suspected POAG patients. Thirty-four suspected POAG participants, from 18 to 73 years old, took part in this study. Foveal and peripheral CS were measured in all suspected POAG participants. The measurement system database has foveal CS values of 71 healthy individuals, from 20 to 69 years old. For our purposes, on one hand, we used these values published by Santillán and colleagues [30] to establish the foveal control group. On the other hand, 28 healthy volunteers, from 23 to 64 years old, were recruited to establish the peripheral control group. Participants from each group were classified by age range (Under and Over 50 y.o.). Foveal and Peripheral CS were tested according to the measurement conditions described in the general methods section.

#### 2.2.2. Experiment 2: Comparison with Early POAG Patients

The purpose of experiment 2 was to assess the significance of foveal CS in suspected POAG participants compared to patients diagnosed with early POAG. Thirty-four early POAG patients, between 20 to 68 years of age, participated in this study. Early POAG participants were age-matched with the POAG-suspect group. Partial results of this cohort (17 participants) were previously reported [38]. Early POAG patients were also grouped by age range (Under and Over 50 y.o.). Foveal CS was tested on the early POAG group according to the measurement conditions described in the general methods section.

## 3. Results

Peripheral CS was assessed on 60 eyes of 34 patients with suspected POAG (data from one eye of ten participants were excluded according to general methods described above) and on 46 eyes of 28 healthy individuals (data from one eye of ten participants were excluded). Foveal CS was assessed on 65 eyes of the suspected POAG group (data from one eye of three participants were excluded) and on 51 eyes of 34 patients with early POAG (data from one eye of thirteen participants were excluded). The foveal control group included 129 eyes of 71 healthy individuals (data from one eye of thirteen participants were excluded).

There was no age-significant difference between the suspected POAG group and: the early POAG group (F (1, 66) = 0.085; *p* = 0.772. Under 50 y.o.: F (1, 17) = 0.002; *p* = 0.962. Over 50 y.o.: F (1, 47) = 0.056; *p* = 0.814), the peripheral control group (F (1, 60) = 0.460; *p* = 0.500. Under 50 y.o.: F (1, 18) = 0.001; *p* = 0.980. Over 50 y.o.: F (1, 40) = 0.447; *p* = 0.508), and the foveal control group (Under 50 y.o.: F (1, 48) = 0.003; *p* = 0.958. Over 50 y.o.: F (1, 53) = 0.040; *p* = 0.842). The age of the foveal control group was not significantly different from the early POAG group (Under 50 y.o.: F (1, 47) = 0.001; *p* = 0.980. Over 50 y.o.: F (1, 54) = 0.002; *p* = 0.963). Table 1 summarizes the clinical characteristics of each group.

### 3.1. Experiment 1: Contrast Sensitivity in Suspected POAG Patients

**Figure 1:**
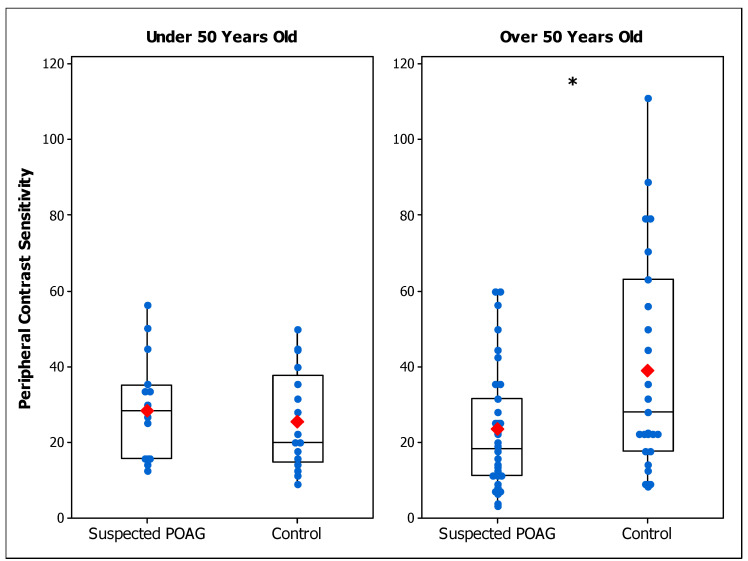
Box plot of peripheral contrast sensitivity in suspected primary open-angle glaucoma (POAG) patients and control participants for those under 50 years old (**left panel**) and over 50 years old (**right panel**). Blue dots are the individual’s values and red marks are the mean values of each group. Differences between groups are indicated as (*) for *p* < 0.05.

**Figure 2:**
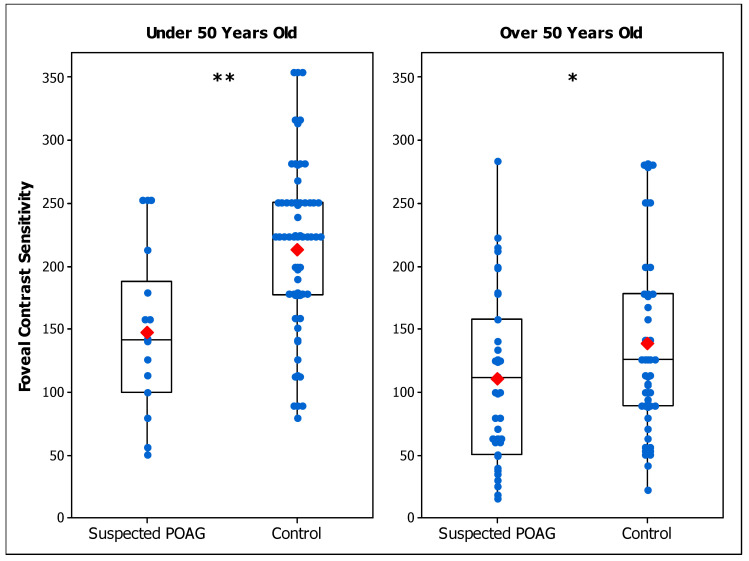
Box plot of foveal contrast sensitivity in suspected primary open-angle glaucoma (POAG) patients and control participants for those under 50 years old (**left panel**) and over 50 years old (**right panel**). Blue dots are the individual’s values and red marks are the mean values of each group. Differences between groups are indicated as (*) for *p* < 0.05, (**) for *p* < 0.01.

Table 2 summarizes the CS results of suspected POAG patients and control participants for each age range in terms of mean and standard deviation values. Figure 1 shows peripheral CS results of suspected POAG and control groups. Peripheral CS was significantly reduced in the POAG-suspect group compared to the peripheral control group for those over 50 years of age (F (1, 40) = 4.48; *p* = 0.040), but not for those under 50 years (F (1, 18) = 0.14; *p* = 0.709). Foveal CS was found to be significantly lower in the POAG-suspect group than in the foveal control group, for both age ranges (Under 50: F (1, 48) = 9.14; *p* = 0.004. Over 50: F (1, 53) = 4.26; *p* = 0.044) (Figure 2).

The results show a lower CS in patients with suspected POAG compared to control groups. Regarding the evaluation in the inferonasal quadrant, CS was found to be reduced only for those over 50 years of age, indicating that the effect of POAG in peripheral CS is age dependent. On the other hand, the foveal assessment shows a reduced CS in patients for both age ranges, indicating that potential early signs of POAG have an effect on CS and this effect is independent of age. Since statistical differences between the POAG-suspect group and the control group were found for both age ranges only for foveal conditions, in Experiment 2 we investigated how important the reduction in foveal CS was for POAG-suspect participants compared to results from patients that were diagnosed as having early POAG.

### 3.2. Experiment 2: Comparison with Early POAG Patients

#### 3.2.1. Foveal CS in Early and Suspected POAG Groups

**Figure 3:**
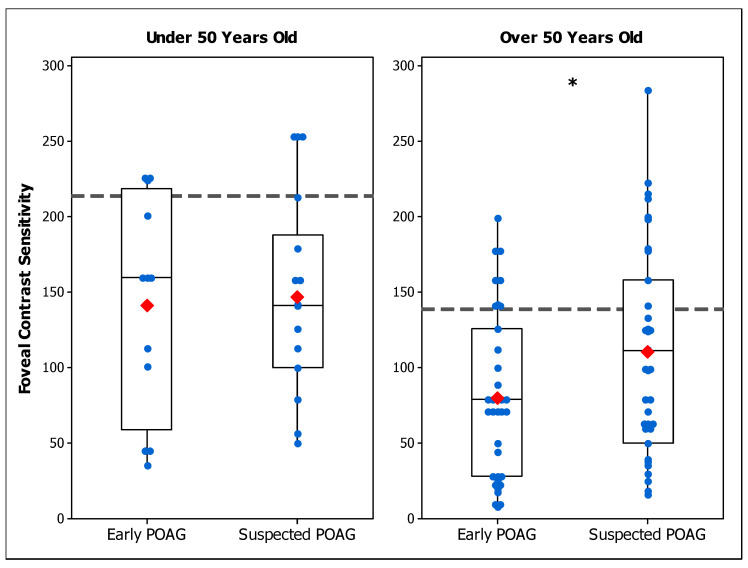
Box plot of foveal contrast sensitivity in early primary open-angle glaucoma (POAG) and suspected POAG patients under 50 years old (**left panel**) and over 50 years old (**right panel**). Blue dots are the individual’s values and red marks are the mean values of each group. The mean foveal control group value is shown by the dashed line. Differences between groups are indicated as (*) for *p* < 0.05.

In Table 2, the CS results of suspected POAG patients and early POAG participants were summarized for each age range in terms of mean and standard deviation values. Figure 3 shows the foveal CS in early POAG and POAG-suspect patients for each age range. An ANOVA showed that foveal CS in the early POAG group is reduced compared to the POAG-suspect group for those over 50 years old (F (1, 47) = 4.38; *p* = 0.042). However, the difference was not statistically significant for patients under 50 years old (F (1, 17) = 0.12; *p* = 0.733). As in our previous study [38], foveal CS was found to be lower in early POAG patients than in the control group for both age ranges (Under 50 y.o.: F (1, 47) = 10.09; *p* = 0.002; Over 50 y.o.: F (1, 54) = 19.60; *p* < 0.001).

The results show a reduced foveal CS in the early POAG group compared to the suspected POAG group when the participants were over 50 years of age. For younger participants, our results show that CS in the suspected POAG group was as reduced as in the early POAG group.

#### 3.2.2. Correlation of Contrast Sensitivity with Clinical Parameters

Pearson correlations were analyzed between CS results and clinical parameters obtained by the eye examination of early and suspected POAG patients. The clinical parameters assessed were Age, VA, IOP, CDR, and MD. Table 3 summarizes the correlation results.

**Figure 4:**
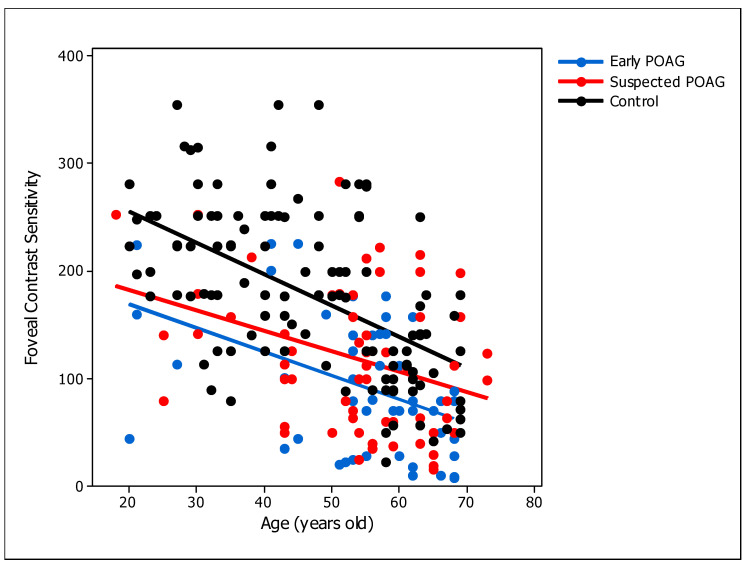
Foveal contrast sensitivity versus age of the participants. Circular dots represent the individual’s values. Solid lines represent the linear model for each group. POAG: primary open-angle glaucoma. The blue color represents the early POAG group (slope = −2.21, intercept = 213.45; r = −0.45; *p* = 0.001). The red color represents the suspected POAG group (slope = −1.9, intercept = 220.17; r = −0.37; *p* = 0.002). The black color represents the control group (slope = −2.91, intercept = 314; r = −0.53; *p* < 0.001). Control data was obtained from the study of Santillán and colleagues [30].

**Figure 5:**
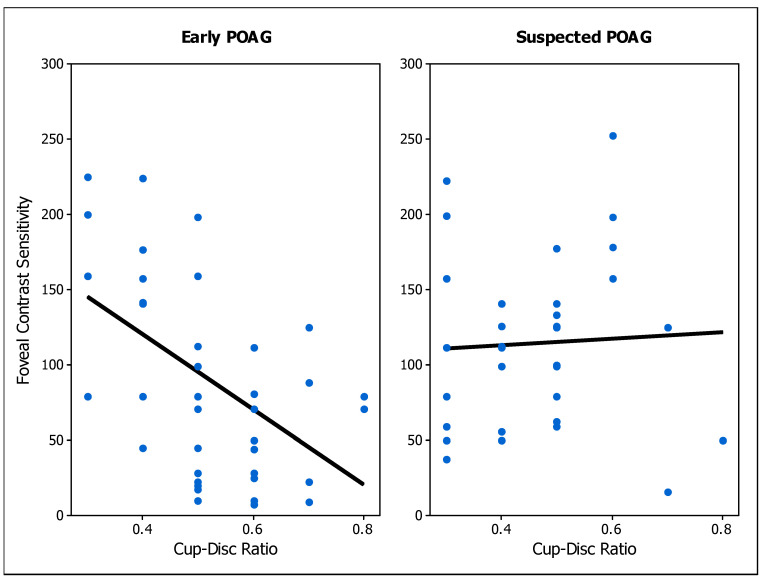
Foveal contrast sensitivity versus cup-to-disc ratio (CDR) in early primary open-angle glaucoma (POAG) (**left panel)** and suspected POAG patients (**right panel**). Circular blue dots represent the individual’s values. Solid black lines represent the linear model for each group (Early POAG group: *p*-value < 0.001. Suspected POAG group: *p*-value = 0.766).

As expected, there was a negative correlation between foveal CS and age [45] (Table 3). This association was weak for the suspected POAG group, and moderate for the early POAG and foveal control groups (Figure 4). CDR is a common measurement used to assess structural damage, therefore we wondered how related CS is with the CDR in the patient groups. There was a negative moderate significant correlation found between CDR and foveal CS of the early POAG group, whereas a negligible correlation was found for the POAG-suspect group (Figure 5).

### 3.3. Assessment of CS as a Screening Tool

The results of Experiments 1 and 2 indicated that CS is affected in both suspected POAG and early POAG patient groups. To evaluate the diagnostic accuracy of CS as a screening tool for discriminating patients from healthy subjects, ROC analyses were performed. Also, we evaluate the ability of CS to differentiate early POAG from suspected POAG patients.

For our purposes, the diagnostic accuracy interpretation was based on the area under the ROC curve (AUC). An AUC ≥ 0.9 is considered excellent, 0.8 ≤ AUC < 0.9 is good, 0.7 ≤ AUC < 0.8 is fair, 0.6 ≤ AUC < 0.7 is poor, and less than 0.6 is a fail [46]. Table 4 summarizes the ROC analysis results for each condition tested.

The ROC analysis shows that peripheral CS, as expected, has failed to differentiate suspected POAG patients from control individuals for the younger cohort (AUC = 0.55; *p* = 0.610). For participants over 50 years old, peripheral CS has poor diagnostic accuracy (AUC = 0.67; *p* = 0.009) [46].

**Figure 6:**
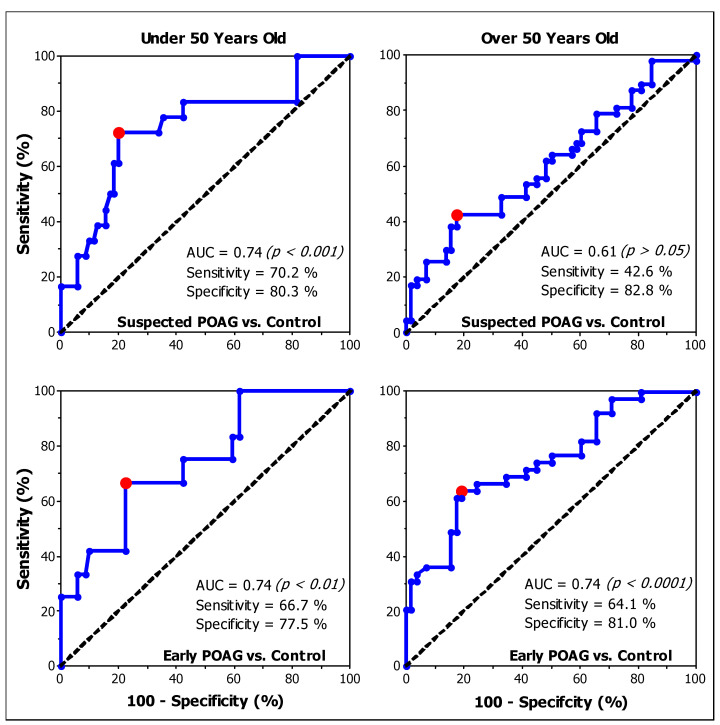
Receiver characteristic operating (ROC) curves for foveal contrast sensitivity. POAG: primary open-angle glaucoma; AUC: area under the curve; Upper panel: suspected POAG patients; Lower panel: early POAG patients; (**Left panel**): Under 50 years old groups; (**Right panel**): Over 50 years old groups. The dotted black lines represent the diagonals with AUC = 0.5 (no discrimination). The red dots represent the pair sensitivity and 100-specificity for the cut-off values. *p*: significance level.

Foveal CS failed in differentiating suspected POAG patients from healthy subjects when participants were over 50 years old (AUC = 0.61; *p* = 0.052) (Figure 6). Instead, for participants under 50, foveal CS has fair diagnostic accuracy (AUC = 0.74; *p* < 0.001). Regarding early POAG patients, the analysis shows that foveal CS has fair diagnostic accuracy for both age ranges (Under 50: AUC = 0.74; *p* = 0.002. Over 50: AUC = 0.74; *p* < 0.0001) (Figure 6).

Finally, foveal CS has poor accuracy in discriminating suspected from early POAG patients for participants over 50 years old (AUC = 0.63; *p* = 0.036). However, for the younger patients, foveal CS failed to discriminate between these two groups of patients (AUC = 0.50; *p* = 0.984).

## 4. Discussion

CS was assessed in patients with suspected primary open-angle glaucoma in different experimental conditions and in comparison with early POAG patients. Peripheral assessment showed low CS for patients over 50 years of age, but not for the younger group, comparing to the healthy age-matched control groups. Foveal CS was lower in POAG suspected patients than in healthy age-matched control participants for both age groups (under and over 50 years old). Similar results were found in early POAG patients, with lower foveal CS for both age groups than the healthy cohort. We found a lower foveal CS for the early POAG group when compared to the POAG suspected group for participants over 50 y.o. However, we did not find a difference between these groups for patients under 50 y.o. Together, these results show how contrast sensitivity is lower in patients with high risk and at early stage of POAG compared to healthy individuals.

Peripheral CS was previously found to be diminished in POAG [25], early to moderate POAG [21], and early POAG patients [29,47], in agreement with our results for suspected POAG subjects. However, in ocular hypertension (OHT) patients CS evaluated at 15° in the temporal retinal quadrant remained the same as in the control group [21]. The CS impairment found in our study at the inferonasal quadrant (9° of eccentricity), suggests that this retinal area could be more sensitive to early visual changes. Indeed, the inferior retina represents a more vulnerable zone to be affected in the early stages of glaucoma [9,48]. In another study involving OHT patients with a high risk of developing POAG, differences with age-matched controls were found at high eccentricities (20° and 25° outside the fovea) but not at lower eccentricities (10° and 15°) at photopic level [25]. Explanations of these spatial differences might include optical aberrations, light-level conditions, and photoreceptor signaling, however, these speculations are out of the scope of the present study.

Regarding foveal CS, several studies have reported low CS in patients with different stages of glaucoma [22,26,27,49,50,51]. However, there is little evidence about CS in previous stages of POAG. Falcão-Reis and colleagues found low foveal CS in POAG patients, although high-risk OHT patients did not present differences with the control group [25], in disagreement with our results in suspected POAG patients. Since they used flickering stimuli while we used static patterns, we can argue that static patterns are more appropriate than flickering ones for detecting CS defects in POAG-suspect patients. Indeed, Ansari and colleagues found a large, but not significant, CS difference between normal and OHT patients with stationary compared to flickering gratings [21]. On the other hand, a trend of decreasing photopic CS with increasing severity of glaucoma has been previously shown [26,28], in agreement with our results in which a lower CS reduction was found in POAG suspected patients compared to early POAG patients.

Considering the spatial frequency, the magnitude of the depression in CS for early POAG patients was reported to be larger at spatial frequencies around the peak contrast sensitivity function than at other spatial frequencies [24,52]. Since, for our experimental conditions, a peak CS was found around 4 c/g for healthy observers [30], the assessment at maximum CS could be more appropriate to evaluate early visual changes caused by POAG.

The structural impairment measured by the CDR was significantly correlated with foveal CS in early POAG patients, in agreement with previous evidence of significant correlations between CS and ganglion cell macular thickness [28] and CDR [26]. This association suggests that CS decreases as the damage to the optic disc increases, and provides further support for our results. However, in POAG-suspect subjects, neither foveal nor peripheral CS were correlated with CDR. Since suspected POAG participants were patients with a risk of developing glaucoma, these results suggest an impairment of CS prior to established optic disc damage. Considering functional visual tests, CS was not significantly correlated with VA and MD. Since VA is the minimum size detectable of a high contrast pattern, it is possible that this function is related to CS measured at higher than intermediate spatial frequencies. On the other hand, correlations between foveal CS and MD have been previously reported in early to advanced glaucoma patients [22,26,28]. As our cohort included only patients with no or mild visual field loss, MD was almost not expected to be associated with CS, suggesting that CS impairment precedes visual field score changes.

The effect of participants’ age on CS has been largely studied [29,30,31,32], demonstrating that older adults have lower CS than younger ones. Our results showed a significant association of foveal CS with age for both early and suspected POAG patients, indicating an effect of age on CS. This effect was similar to the one found for the control group, in agreement with the literature (Figure 4). Each patient group (early and suspected) was age-matched with the control group, implying that the low CS found in these patients is mainly caused by visual deficits of optic neuropathy, and not because of the participants’ age. Interestingly, foveal CS of POAG suspected patients was more similar to early POAG results for young than for older participants (Figs. 3 and 4), suggesting that CS might be more informative at a young age. However, this trend was not found for peripheral conditions, where CS was only low in POAG-suspect group for participants over 50 years of age, suggesting that peripheral CS is not informative for a young cohort.

Diagnosing POAG at its early stages continues to be a challenge [53,54]. Optical coherence tomography has improved the assessment of early structural damage in suspected POAG patients [9,55]. Assessment of the visual field has been demonstrated to be adequate for diagnosing and assessing the progression of POAG at more advanced stages [13,56]. Other functional techniques, such as chromatic pupillometry [57,58] and electroretinography [59,60], were also used to evaluate functional changes in suspected POAG. Studies using these techniques reported an early deficit in ganglion cell function in POAG suspects [61,62,63]. There is wide evidence of low CS in POAG patients. Our study provides evidence that such performance occurs also in POAG-suspect eyes and can be revealed in visual function, even when the visual field is within normal values and the VA is preserved.

Although CS is a potential measure for exploring the visual system, it has not been broadly implemented in clinical settings, possibly because faster tests are preferred. Our protocol for measuring peripheral and foveal CS was relatively long duration in assessing both eyes (approximately 30 min including adaptation periods). Instead, we propose a reduced protocol, measuring foveal CS (photopic, 4 c/g), lasting about 10 min to test both eyes and also being appropriate to examine patients in a wide age range. In addition, low values of CS were found for patients with other major ocular affections, such as cataracts [64], age-related macular degeneration [65], and diabetic retinopathy [66], which are leading causes of blindness and visual impairment [67]. Therefore, values beyond the normal curve could reveal different factors, and diagnosis needs to rely on other clinical tests. It shows that further work needs to be done regarding the discriminative power of CS.

Considering individual values, there was an overlap between patients and healthy controls (Figure 1, Figure 2 and Figure 3). The ROC analysis demonstrated that, for most of the conditions, CS does not have good diagnostic accuracy to differentiate suspected POAG patients from healthy individuals [46,68] (Table 4), nor to discriminate between early and suspected POAG patients. The low sensitivities found in the conditions evaluated indicate that a large number of suspected or early POAG patients would pass the test as healthy controls. The efficacy of CS for detecting POAG was reported to be higher at more advanced stages [51], while in early POAG patients [50,52] a poor diagnostic accuracy was found, in agreement with our results. However, our study presents evidence that CS is affected in a group of patients suspected of having POAG and at early stages of POAG. Thus, CS may serve as a test to help clinicians to make a final diagnostic decision.

### Study Limitations

Our measurements considered only one spatial frequency (4 c/d). We know from previous results that this frequency was the most informative in early glaucoma patients [24,30,52], but other frequencies may provide information about different aspects of spatial vision [69]. Investigation of the other spatial frequencies was beyond the scope of this study. A second limitation of this study is that our cohort of suspected patients may never develop POAG because they are in a preclinical stage. A longitudinal study could unveil how important the results of this cross-sectional study are concerning the progression of the disease. A more complete characterization of the retina could have been achieved with an OCT device; however, this technology was not available in the clinical settings where this study was conducted. Since this limitation is common in the developing world [70], our study employed the clinical assessment tools available in the place of our study.

## 5. Conclusions

Our findings provide evidence for the valuable role of CS in providing information about visual deficits in a group of suspected POAG patients. However, CS did not provide good discrimination at the individual level. Our results also suggest that retinal function may be affected before structural damage even in patients at risk for developing primary open-angle glaucoma. Therefore, spatial vision may be impaired in the early stages of POAG. These findings highlight the importance of developing further work aimed at testing spatial vision testing tools with greater power and which are appropriate for clinical settings.

## Figures and Tables

**Table 1:** Clinical characteristics of each group. POAG: primary open-angle glaucoma; F: female; M: male; PA: peripheral assessment; FA: foveal assessment; VA: visual acuity; IOP: intraocular pressure; MD: mean deviation; CDR: cup to disc ratio; SD: standard deviation.

	Suspected POAG Group	Early POAG Group	Peripheral Control Group	Foveal Control Group
N° Participants, Gender	34 20 F, 14 M	34 21 F; 13 M	28 12 F; 16 M	71 34 F; 37 M
Age, mean ± SD (years old)	52 ± 13	53 ± 13	50 ± 13	46 ± 14
N° Under 50 y.o. (n° of eyes)	10 (16 PA, 18 FA)	9 (12 FA)	10 (17 PA)	40 (71 FA)
Age, mean ± SD	35 ± 9	35 ± 12	35 ± 8	35 ± 8
N° Over 50 y.o. (n° of eyes)	24 (44 PA, 47 FA)	25 (39 FA)	18 (29 PA)	31 (58 FA)
Age, mean ± SD	59 ± 6	60 ± 5	58 ± 5	60 ± 6
VA, mean ± SD (logMAR)	−0.09 ± 0.10 ^a^	−0.09 ± 0.10 ^a^	0.05 ± 0.10	0.05 ± 0.10
IOP, mean ± SD (mmHg)	15 ± 3	14 ± 3	15 ± 2	15 ± 2
MD, mean ± SD	0.01 ± 1.7	0.2 ± 1.6 ^a^	−1.2 ± 2.0	−1.2 ± 2.0
CDR, mean ± SD	0.4 ± 0.1 ^a,b^	0.5 ± 0.1 ^a^	0.2 ± 0.1	0.2 ± 0.1

^a^ Statistically significant difference in comparison to the control groups found. ^b^ Statistically significant difference found in comparison to the early PAOG group found.

**Table 2:** Contrast sensitivity results in under 50 years old and over 50 years old participant groups (mean ± standard deviation values). POAG: primary open-angle glaucoma; CS: contrast sensitivity.

	Suspected POAG Group	Early POAG	Control Group
Peripheral CS Under 50	28.3 ± 13.7	-	25.6 ± 13.1
Peripheral CS Over 50	23.4 ± 16.1 ^a^	-	39.1 ± 28.2
Foveal CS Under 50	146.8 ± 63.3 ^a^	141.2 ± 72.6 ^a,b^	213.5 ± 66.2
Foveal CS Under 50	110.5 ± 65.0 ^a^	80.2 ± 54.5 ^a,b^	138.6 ± 71.7

^a^ Statistically significant difference in comparison to the control group found. ^b^ Statistically significant difference in comparison to the suspected POAG group found.

**Table 3:** Pearson correlation between contrast sensitivity and clinical parameters. CS: contrast sensitivity; POAG: primary open-angle glaucoma; VA: visual acuity; IOP: intraocular pressure; CDR: cup-to-disc ratio; MD: mean deviation of the visual field; r: Pearson coefficient; *p*: significance level.

	Age	VA	IOP	CDR	MD
Early POAG Foveal CS	r = −0.45 (*p* = 0.001)	r = 0.21 (*p* = 0.178)	r = 0.24 (*p* = 0.129)	r = −0.53 (*p* < 0.001)	r = 0.09 (*p* = 0.674)
POAG Suspect Foveal CS	r = −0.37 (*p* = 0.002)	r = 0.24 (*p* = 0.076)	r = −0.11 (*p* = 0.446)	r = 0.05 (*p* = 0.766)	r = −0.14 (*p* = 0.527)
POAG Suspect Peripheral CS	r = −0.21 (*p* = 0.106)	not applicable	r = −0.23 (*p* = 0.116)	r = −0.12 (*p* = 0.515)	r = −0.10 (*p* = 0.649)

**Table 4:** Receiver operating curves analysis results of contrast sensitivity in suspected and early primary open-angle glaucoma (POAG) groups. AUC: area under the curve; CI: confidence interval.

	SC	Cut Off Value	Sensitivity (%)	Specificity (%)	AUC	AUC 95% CI	Interpretation
Suspected POAG vs. Control	Peripheral (under 50)	22.3	62.5	58.8	0.55	0.37–0.73	Fail
	Peripheral (over 50)	22.3	56.8	72.4	0.67	0.55–0.78	Poor
	Foveal (under 50)	158.1	72.2	80.3	0.74	0.64–0.83	Fair
	Foveal (over 50)	79.3	42.6	82.8	0.61	0.52–0.70	Fail
Early POAG vs. Control	Foveal (under 50)	159.6	66.7	77.5	0.74	0.64–0.83	Fair
	Foveal (over 50)	80.9	64.1	81.0	0.74	0.65–0.83	Fair
Early vs. Suspected POAG	Foveal (under 50)	158.1	58.3	72.2	0.50	0.32–0.69	Fail
	Foveal (over 50)	28.1	30.8	93.6	0.63	0.52–0.73	Poor

## Data Availability

The authors will make the anonymized data supporting the conclusions available upon request.
